# Intra-articular platelet-rich plasma injection for knee osteoarthritis: a summary of meta-analyses

**DOI:** 10.1186/s13018-019-1363-y

**Published:** 2019-11-27

**Authors:** Pu Chen, Liuwei Huang, Yufeng Ma, Dong Zhang, Xiaozhe Zhang, Jun Zhou, Anmin Ruan, Qingfu Wang

**Affiliations:** 1 Beijing University of Chinese Medicine, Beijing, China; 2 Southern Medical University, Guangzhou, Guangdong Province China; 3 Department of Orthopaedic Surgery, Beijing University of Chinese Medicine Third Affiliated Hospital, No. 51, XiaoGuan street, AnDing gate, ChaoYang district, Beijing, China

**Keywords:** Platelet-rich plasma, Knee osteoarthritis, Intra-articular injection, Meta-analysis, Hyaluronic acid, Placebo

## Abstract

**Objective:**

The purpose of this study was (1) to perform a summary of meta-analyses comparing platelet-rich plasma (PRP) injection with hyaluronic acid (HA) and placebo injection for KOA patients, (2) to determine which meta-analysis provides the best available evidence to making proposals for the use of PRP in the treatment of KOA patients, and (3) to highlight gaps in the literature that require future investigation.

**Material and methods:**

PubMed, EMBASE, and Cochrane databases search were performed for meta-analyses which compared PRP injection with HA or placebo. Clinical outcomes and adverse events were extracted from these meta-analyses. Meta-analysis quality was assessed using the Quality of Reporting of Meta-analyses (QUOROM) systems and the Oxman-Guyatt quality appraisal tool. The Jadad decision algorithm was also used to determine which meta-analysis provided the best available evidence.

**Results:**

Four meta-analyses were included in our study, and all of these articles were Level I evidence. The QUOROM score of each included meta-analysis range from 14 to 17 points (mean score 15, maximum score 18), and the Oxman-Guyatt score range from 4 to 6 points (mean score 5, maximum score 7). Three meta-analyses indicated PRP showed more benefit in pain relief and functional improvement than the control group, and the other one suggested no difference between these groups. All included meta-analyses found no statistical difference in adverse events between these groups. In addition, a meta-analysis conducted by Shen et al. got the highest methodological quality score and suggested that PRP provided better pain relief and function improvement in the treatment of KOA.

**Conclusions:**

For short-term follow-up (≤1 year), intra-articular PRP injection is more effective in terms of pain relief and function improvement in the treatment of KOA patients than HA and placebo, and there is no difference in the risk of an adverse event between PRP and HA or placebo.

**Level of evidence:**

Level I evidence, a summary of meta-analyses

**Trial registration:**

PROSPERO ID CRD42018116168

## Introduction

Knee osteoarthritis (KOA) is one of the most common degenerative joint diseases with continuous pain and loss of function [1] and characterized by progressive loss of articular cartilage, inflammation of synovial membrane, and changes in the bones under the cartilage [2–5]. It was reported that among older adults, the risk of lower limb disability caused by KOA is at least 40% [6], and KOA is considered as one of top ten causes of disability [7]. To date, however, there are no treatment methods that can reverse or alter the progression of KOA. Although total knee arthroplasty (TKA) is regarded as the last choice if osteoarthritis progresses to end-stage [8], there is a significant risk of complications including revision, infection, and unsatisfied function [8–10]. Therefore, in terms of the younger and middle-aged patients of KOA, non-surgical interventions attract more and more attention, including physical therapy, oral nonsteroidal anti-inflammatory drugs (NSAIDs), hyaluronic acid (HA), ozone, and corticosteroids injection [8, 11].

In the last 10 years, growth factors aroused people’s interest for its properties of repair tissue lesion and maintain normal tissue structure, especially platelet-rich plasma (PRP) injection [12–15]. PRP contains a high concentration of platelets, which are obtained by centrifugation of autologous blood [16]. Various growth factors and cytokines are released after the degranulation of platelets and to accelerate cartilage matrix synthesis, restrain synovial membrane inflammation, and promote cartilage healing [17, 18]. Owing to the properties of regenerative effect and anti-inflammatory potential, PRP is widely used in musculoskeletal diseases, such as rotator cuff tear, lateral epicondylitis, patellar tendinopathy, osteoarthritis [19–26].

Lots of articles [27–36] compared the clinical outcomes of intra-articular PRP injection with other conservative treatment methods (including oral NSAIDs, HA, and corticosteroids injection), and there are different results among these comparisons. The American Academy of Orthopaedic Surgeons Clinical Guidelines suggested that HA injection is not recommended for the treatment of KOA, while PRP injection is “not recommend for or against” [8]. The OA Research Society International (OARSI) Guidelines [37] provide an “uncertain” recommendation for HA injection in the treatment of KOA, while do not mention the PRP injection. Meanwhile, Campbell [34] conducted a systematic review of overlapping meta-analyses, suggested that PRP injection may increase the local adverse reactions than HA. However, several meta-analyses [30–33, 35] published in the last 3 years indicated PRP injection does not have more adverse events than HA injection.

Therefore, the purpose of this study was (1) to perform a summary of meta-analyses comparing PRP injection with HA injection for KOA patients, (2) to determine which meta-analysis provides the best available evidence to making proposals for the use of PRP in the treatment of KOA patients, and (3) to highlight gaps in the literature that require future investigation. We hypothesized that PRP injection is more effective in the treatment of KOA patients and with a similar risk of adverse events than HA and placebo.

## Methods

### Literature search

The PubMed, EMBASE, and Cochrane database was searched to perform a summary of meta-analysis according to the Preferred Reporting Items for Systematic Reviews and Meta-Analysis (PRISMA) statement [38]. It has been registered in PROSPERO. The lasted literature search was conducted on November 12, 2018. The following search terms were used: [platelet-rich plasma OR PRP] AND [knee arthritis OR arthritis OR knee osteoarthritis OR osteoarthritis] AND [meta-analysis OR systematic review]. At the same time, the citations of the included meta-analyses were evaluated to see if there were any suitable literatures for inclusion. When necessary, the corresponding author of the study was contacted for further information.

### Inclusion criteria

The inclusion criteria are as follows:

1. Compared the outcomes of intra-articular platelet-rich plasma (IA-PRP) injection with intra-articular hyaluronic acid (IA-HA) or placebo injection
2. The meta-analysis of randomized controlled trials
3. Clinical research
4. Written in English
5. Published after the year 2000

### Exclusion criteria

The exclusion criteria are as follows:

1. Did not compare the outcomes of intra-articular platelet-rich plasma (IA-PRP) injection with intra-articular hyaluronic acid (IA-HA) or placebo injection
2. Meta-analysis included non-randomized controlled trials
3. Cadaveric, animal, or biomechanical research
4. Not written in English
5. Published before the year 2000
6. Network meta-analysis or overlapping meta-analysis

### Quality appraisal

Each included study was evaluated with the Quality of Reporting of Meta-analyses (QUOROM) system [39]. It is divided into 6 headings, and 18 items totally, including searching, validity assessment, data abstraction, trial flow, study characteristics, and so on. The Oxman-Guyatt quality appraisal tool [40] was also used to assess the quality of meta-analysis. Two trained reviewers assessed the included meta-analysis respectively. And the final decision was made by a third author after which reviewed the article if they have different opinions. Moreover, the bias was noted while it was reported by individual trials in the literature. In addition, three authors used the Jadad decision algorithm [41] to guide interpretation of discordant reviews respectively, and the results determined which of the included systematic reviews provided the highest quality current evidence to make recommendations for knee osteoarthritis.

### Data extraction and statistical analysis

Data were extracted by two trained authors from the included articles. It included the following data: author, the year of publication, level of evidence included in the studies, the searched databases, eligibility criteria, no. of included articles, no. of patients, basic patient information, time of follow-up, adverse events, patient satisfaction. And the following standardized outcome scores were collected: visual analog scale (VAS) pain score, Western Ontario and McMaster Universities Osteoarthritis Index pain (WOMAC) score, International Knee Documentation Committee (IKDC) score, and Lequesne index.

In addition, we also recorded the following characteristics of each included systematic review: the rationale for repeating the systematic review, the number of previous systematic reviews actually cited compared with maximum number that could possibly have been cited in each study, the search methodology of each included study, the demographic data and characteristics of the review, the heterogeneity and subgroup analyses of primary studies in the systematic review, and the conclusions of the review regarding whether IA-HA was more clinically effective in terms of pain relief, functional scores, and side effects.

## Results

### Literature search results

**Fig. 1:**
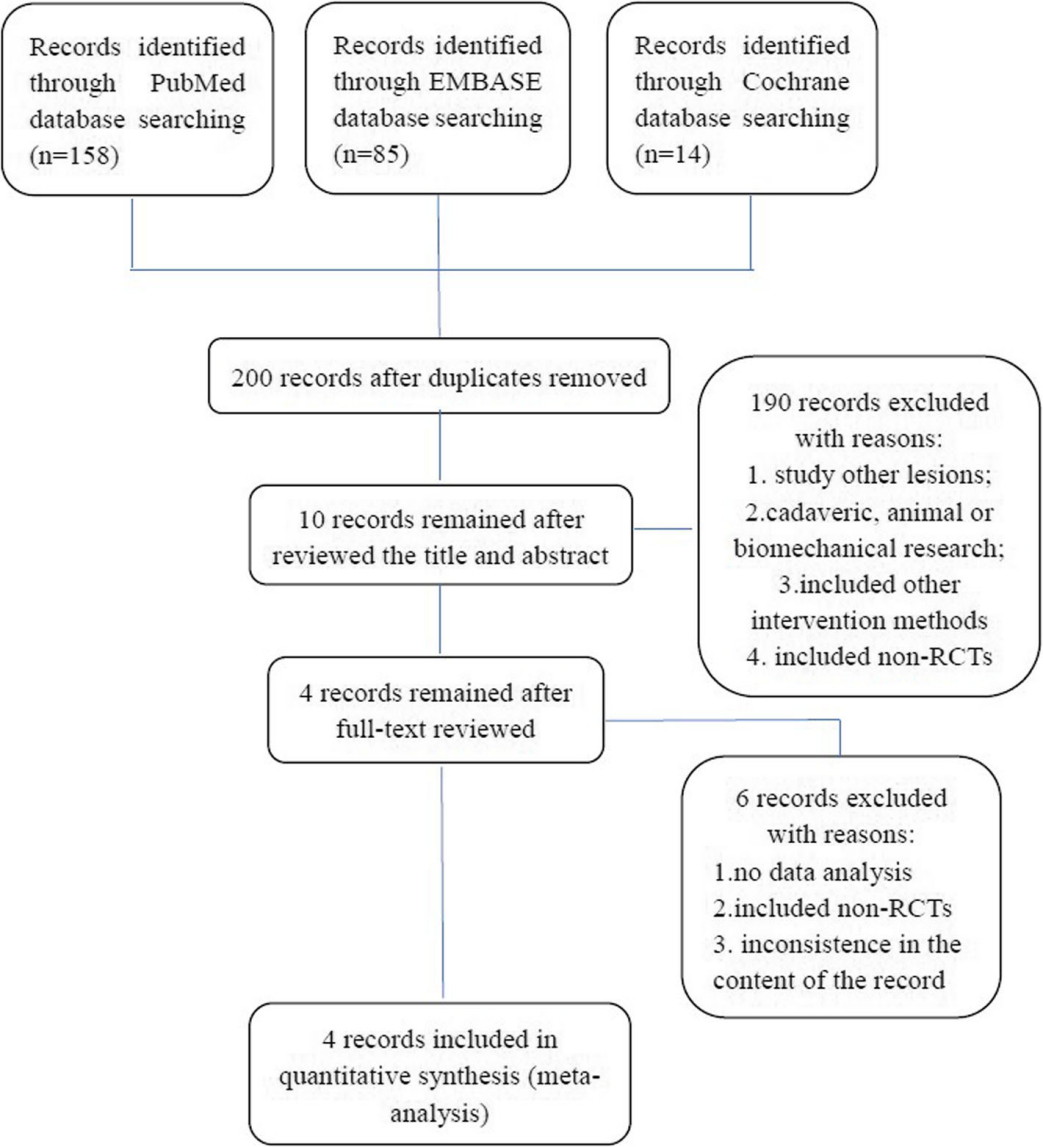
The PRISMA flow diagram

There were 200 articles that remained after removal of duplicates, only 4 studies [30–33] met our inclusion and exclusion criteria and were included in the summary of meta-analysis (Fig. 1). All included articles were recently published (between 2016 and 2017). Each study only included randomized controlled trials and was Level I evidence article. All included studies had no conflict of interest. Among these meta-analyses, three articles [30, 31, 33] compared the outcomes of PRP injection with HA, corticosteroids, ozone, or placebo at 6 and 12 months follow-up; two studies [31, 33] also compared the outcomes at 3 months follow-up; one paper [32] compared at less than or equal to 12 months follow-up; and all of these meta-analyses were short-term follow-up.

### Authors’ assessment of prior systematic review literature

None of these included articles cited all of the prior systematic reviews or meta-analyses that were possible to be obtained at the time of publication, and two articles [32, 33] cited more than half of prior systematic reviews or meta-analyses (Table 1). Meanwhile, only Shen et al. [33] was registered in PROSPERO (CRD42016045410), other articles did not enroll in the Cochrane or PROSPERO website.

**Table 1:** Number of systematic reviews or meta-analyses actually cited compared with maximum number that could possibly have been cited, and the rationale for repeating the systematic review or meta-analyses

	Date of publication	Date of last literature search	Possible to cite*	Cited^#^	Cochrane or PROSPERO register	Rationale for repeating meta-analysis as extracted from article
Kanchanatawan et al.	19 Sep. 2015	13 Aug. 2015	10	7	–	All of the meta-analyses did not strictly pool outcomes from studies of high methodological quality (RCTs) as there were very few RCTs available for review at the time. Sources of heterogeneity were also not assessed. Additional RCTs have since been published. Therefore, we conducted a systematic review and meta-analysis comparing clinical outcomes when treating KOA by PRP injection as compared to HA or placebo.
Dai et al.	22 Sep. 2016	30 Apr. 2016	12	4	–	To date, PRP-preparation techniques, platelet count, number of injections, the use of anticoagulants, activating agents, and severity of OA have varied considerably among studies. Studies reporting the effect of PRP injection in patients with knee OA convey conflicting results. In addition, because of small sample sizes, these studies were not powered adequately to detect the effect of PRP for patients with knee OA.
Xu et al.	11 Nov. 2017	13 May 2016	13	4	–	Previous systematic reviews conducted on the viability of PRP and HA came to the unanimous conclusion that PRP was more effective than HA, but the reliability of this conclusion was more or less affected by inappropriate study selection strategies, incorrect statistical methods, and/or a limitation in the number of included trials. Recently, several new high-quality RCTs had turned out results that are in contrast with those of the previous RCTs and reviews.
Shen et al.	16 Dec. 2017	15 Nov. 2016	15	11	+	Considering that prior reviews either included non-RCTs or only synthesized a small number of RCTs (less than 9) for analysis and that quite a few more RCTs recently have been published, we believe that it is necessary to perform an updated systematic review and meta-analysis, if appropriate, to evaluate whether the evidence-based support for PRP treatment will be strengthened or compromised.

*No. of systematic reviews or meta-analyses possible to cite

^#^No. of systematic reviews or meta-analyses cited

All included meta-analysis had demonstrated the rationale for repeated meta-analysis. The main reasons are as follows: the reliability of pre-existing systematic reviews was more or less affected by inclusion of non-RCTs or a small number of RCTs, more RCTs were published recently, sources of heterogeneity were also not assessed, and small sample sizes were not powered adequately to detect the effect of PRP for patients with KOA (Table 1).

### Outcome measures

Heterogeneity existed in these 4 articles about the standardized and non-standardized patient outcome measures they reported (Table 2). In addition, heterogeneity also existed as follows: leukocyte-rich PRP (LR-PRP) or leukocyte-poor PRP (LP-PRP), single or double spinning, activation or not, PRP injection dose, times, and intervals.

**Table 2:** Outcomes reported by each included study

	Kanchanatawan et al.	Shen et al.	Xu et al.	Dai et al.
Clinical scores				
VAS pain score	+	–	+	–
IKDC score	+	–	+	+
WOMAC total score	+	+	+	+
WOMAC pain score	+	+	–	+
WOMAC function score	+	+	–	+
Lequesne score	+	–	+	+
Patient satisfaction	–	–	–	–
Adverse events	+	+	–	+

All included studies [30–33] used WOMAC total score to compare the outcomes between PRP injection and HA or placebo; WOMAC pain score, WOMAC function score, and adverse events were evaluated in three articles [30, 32, 33]; IKDC score and Lequesne score were also appraised in three articles; and two studies [31, 32] analyzed the VAS pain score. Only one paper [30] performed subgroup analyses which were based on times of PRP injection, PRP spinning techniques, mean platelet concentration, PRP category, activation or not, and risk of bias. Otherwise, none of the included articles assessed patient satisfaction (Table 2).

### Search methodology

Although all studies that met our inclusion criteria searched more than 3 databases for data extraction, there was heterogeneity in the specific databases that were used (Table 3). These included databases are as follows: PubMed, MEDLINE, EMBASE, Cochrane, Scopus, Ovid, and other databases.

**Table 3:** Search methodology used by each included study (*Ovid including EMBASE, EBW reviews, and Cochrane library)

	PubMed	MEDLINE	EMBASE	Cochrane	Scopus	Ovid*	Other	No. of primary studies	RCT
Kanchanatawan et al.	+	+	–	–	+	–	–	9	+
Dai et al.	+	–	+	+	+	–	–	10	+
Xu et al.	–	+	–	–	–	+	–	10	+
Shen et al.	+	–	+	+	+	–	–	14	+

Every included meta-analysis was only selected randomized controlled trial and was a Level I evidence article. Table 4 shows the primary studies which were included in these meta-analyses. The number of primary articles included in these meta-analyses ranges from 9 to 14, and the total number of primary articles was 16. And all primary articles were published in the last 8 years (from 2011 to 2016).

**Table 4:** Primary studies included in meta-analyses

	Kanchanatawan et al.	Shen et al.	Xu et al.	Dai et al.
Cerza F 2012 [42]	+	+	+	+
Li M 2011 [43]	–	+	+	–
Patel et al. [27]	+	+	+	+
Sanchez et al. [28]	+	+	+	+
Vaquerizo V 2013 [44]	+	+	+	+
Filardo et al. [29]	+	+	+	+
Duymus TM 2016 [45]	–	+	+	+
Forogh B 2016 [46]	–	+	–	–
Gormeli G 2015 [47]	+	+	+	+
Raeissadat SA 2015 [48]	+	+	+	+
Smith PA 2016 [49]	–	+	+	+
Montanez-Heredia E 2014 [50]	–	+	–	–
Paterson KL 2016 [51]	–	+	–	+
Spakova T 2012 [52]	–	+	–	–
Filardo G 2012 [53]	+	–	–	–
Rayegani SM 2014 [54]	+	–	–	–

### Study results

The number of patients in these included meta-analyses varied from 1069 to 1423(Table 5), and a total of 1677 patients were included in this summary of meta-analysis. All included meta-analysis did not provide the mean age; we extracted the mean age from prior RCTs which were cited by these meta-analyses, and it ranges from 46.6 to 66.5 years. BMI was indicated in three studies [30, 32, 33], and all of the mean BMI was 24 at least. None of the included studies calculated the mean OA grade (including Kellgren-Lawrence grade and Ahlback grade) and the duration time.

**Table 5:** Demographic data and characteristics of included meta-analyses

	No. of patients	No. of PRP	No. of control	Age (years)	OA grade	Duration time	BMI (kg/m^2^)	Follow-up time (months)	QUOROM score	Oxman-Guyatt score	Conclusion
Kanchanatawan et al.	1175	608	HA: 465 Placebo: 71 Other: 31	52.7–66.4	NR	NR	26–30.9	≤ 12	14	4	For short-term outcomes (≤ 1 year), PRP injection has improved functional outcomes (WOMAC total scores, IKDC, and VAS score) when compared to HA and placebo, but no difference in adverse events.
Dai et al.	1069	562	HA: 429 Placebo: 78 Other: 0	46.6–66.5	NR	NR	25.8–31.0	6, 12	15	5	At 1-year follow-up, PRP injection may have more benefit in pain relief and functional improvement and did not increase the risk of adverse events when compared with HA and placebo in patients with symptomatic KOA.
Xu et al.	1184	594	HA: 465 Placebo: 86 Other: 39	46.6–66.5	NR	NR	NR	3, 6, 12	15	5	PRP was found effective to relieve pain and improve self-report function of patients having knee OA, with a satisfactory level observed for at least 6 months follow-up, but no superiority was observed in its effectiveness when compared with HA.
Shen et al.	1423	718	HA: 563 Placebo: 86 Other: 63	49.9–66.5	NR	NR	24–30.9	3, 6, 12	17	6	Intra-articular PRP injections probably are more efficacious in the treatment of KOA in terms of pain relief and self-reported function improvement at 3, 6, and 12 months follow-up, compared with other injections, including saline placebo, HA, ozone, and corticosteroids.

#### PRP injection versus HA injection

Four articles [30–33] used WOMAC total score to compare the outcomes between PRP and HA, and three articles [31–33] indicated PRP injection was more efficacious than HA injection. However, Dai et al. [30] found only at 12 months follow-up, PRP injection was superior to HA injection, and there was no statistical difference between two intra-articular injection techniques at 6 months follow-up; Xu et al. [31] demonstrated PRP injection showed no superiority than HA when high-quality double-blind RCTs were included merely.

Three studies evaluated the WOMAC pain and function score after PRP and HA injection, Shen et al. [33] showed PRP injection was more effective to reduce pain and improve self-report function than HA injection in KOA patients. In contrast, Kanchanatawan et al. [32] stated that there was no statistical difference between two injection techniques, and Dai et al. [30] indicated PRP was similar to HA at 6 months follow-up but was superior than HA at 12 months.

In terms of VAS, IKDC, and Lequesne score, Kanchanatawan et al. [32] indicated that there was a statistical difference between PRP and HA in VAS and IKDC score, with no difference in Lequesne score; and Xu et al. [31] found no difference in VAS, IKDC, and Lequesne score. Otherwise, Dai et al. [30] demonstrated a statistical difference in IKDC and Lequesne score at 6 months follow-up but showed PRP was superior at 12 months follow-up.

Three meta-analyses [30, 32, 33] assessed the adverse events after intra-articular PRP and HA injection. None of them showed that PRP injection has a higher risk of adverse events than HA injection, and all of them supported that there was no statistical difference in adverse events between two groups.

#### PRP injection versus placebo

All included meta-analyses [30–33] indicated that there was a significant difference between PRP injection and placebo group in clinical outcomes (including WOMAC total, pain, function score, IKDC score, and Lequesne score). And there was no statistical difference in adverse events between the two groups.

Dai et al. [30] also performed a subgroup analyses for WOMAC pain and function scores which were based on times of PRP injection, PRP spinning techniques, mean platelet concentration, PRP category, activation or not, and risk of bias. It suggested that the WOMAC pain and function scores of HA injection were better than PRP injection at 6 months follow-up when the mean platelet concentration was bigger than 5*baseline, LR-PRP, and using activator.

### Study quality and validity

Each included meta-analyses was assessed by the QUOROM score (the maximum possible score is 18), and the score range from 14 to 17 points, with a mean score of 15 **(**Table 5). All included meta-analyses were also evaluated with the Oxman-Guyatt score (the maximum possible score is 7), and the score ranges from 4 to 6 points, with a mean score of 5. And the study was considered to have major flaws when the Oxman-Guyatt score was less than 3 points **(**Table 5).

### Heterogeneity assessment

Several methods were used to assess study heterogeneity, all included meta-analyses [30–33] performed a statistical heterogeneity analysis, and each of these studies also assessed the primary study quality (Table 6). Otherwise, several meta-analyses performed subgroup or sensitivity analysis assessing parameters such as numbers of PRP injection, PRP spinning technique, mean platelet concentration, LP-PRP or LR-PRP, activator or not, the different clinical outcome scores, and adverse events. None of these articles analyzed the influence of age, gender, and OA grade.

**Table 6:** Heterogeneity and subgroup analyses of each included study

	Kanchanatawan et al.		Dai et al.		Xu et al.		Shen et al.
	PRP v HA	PRP v placebo	PRP v HA	PRP v placebo	PRP v HA	PRP v placebo	PRP v Control
Statistical heterogeneity analysis	+		+		+		+
Subgroup or sensitivity analysis							
Primary study quality	+		+		+		+
Age	–		–		–		–
Gender	–		–		–		–
OA grade	–		–		–		–
WOMAC total score	+ (≤ 1 year)	+ (≤ 1 year)	+ (6, 12 months)	+(6, 12 months)	+ (3, 6, 12 months)	+(6 months)	+(3, 6, 12 months)
WOMAC pain score	+ (≤ 1 year)	+ (≤ 1 year)	+ (6, 12 months)	+(6, 12 months)	–	–	+(3, 6, 12 months)
WOMAC functional score	+ (≤ 1 year)	+ (≤ 1 year)	+ (6, 12 months)	+(6, 12 months)	–	–	+(3, 6, 12 months)
Lequesne score	+ (≤ 1 year)	–	+ (6, 12 months)	–	+ (6 months)	–	–
IKDC score	+ (≤ 1 year)	–	+ (6, 12 months)	–	+ (6 months)	+(6 months)	–
VAS score	+ (≤ 1 year)	–	–	–	+ (6 months)	–	–
Adverse events	+	+	+	+	–	–	+
No. of PRP injection (1 or ≥ 2); PRP spinning approach (single or double); mean platelet concentration (> or < 5*baseline); LP or LR PRP; with an activator or not, risk of bias	–		+		–		–

### Application of Jadad decision algorithm

**Fig. 2:**
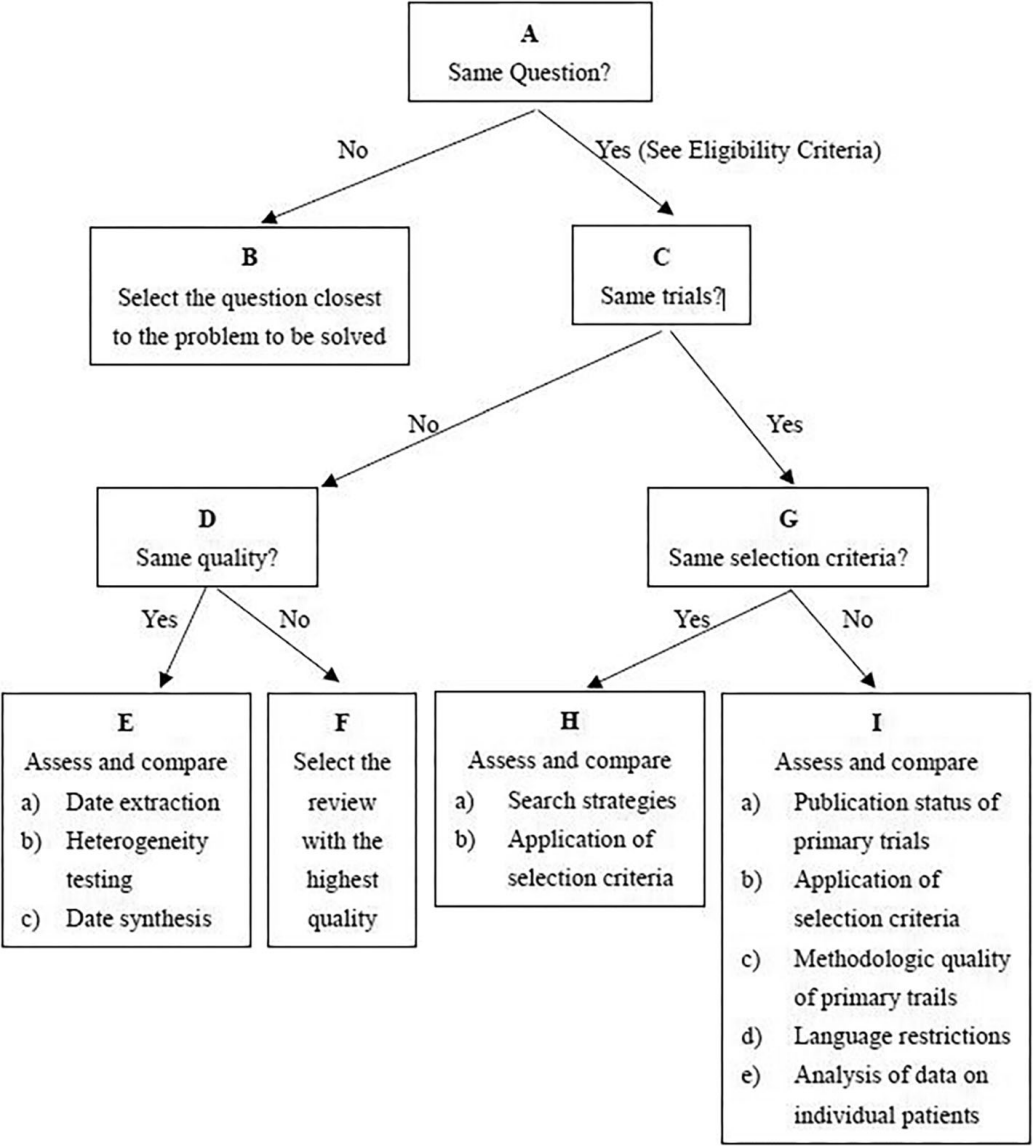
Flow diagram of Jadad decision algorithm

Which of the 4 included meta-analyses offered the best available evidence to making proposals for the use of PRP in the treatment of KOA patients was investigated following the Jadad decision algorithm [41]. Figure 2 shows the flow diagram of the Jadad decision algorithm. Two trained authors selected the same route through the Jadad decision algorithm respectively. Differences between the two authors were resolved by consensus and discussion with a third author. Given that (1) each of the included meta-analyses did not investigate the same question (Tables 2 and 6), (2) did not include the same prior articles (Tables 3 and 4), and (3) have different selection criteria, the Jadad decision algorithm suggests that the best available evidence should be selected based on the publication characteristics and the methodology of primary trials, the language restrictions, and whether analysis of data on individual patients was included in the study. Therefore, the meta-analysis [33] which was conducted by Shen et al. got the highest methodological quality score, with included 14 RCTs, 11 of 15 pre-existing systematic reviews or meta-analyses were cited, and the QUOROM score and Oxman-Guyatt score were 17 and 6, respectively. This meta-analysis indicated that compared with other injections, including saline placebo, HA, ozone, and corticosteroids, intra-articular PRP injection probably is more effective in terms of pain relief and function improvement at 3, 6, and 12 months follow-up in the treatment of KOA patients.

## Discussion

This summary of meta-analyses suggested that based on the best currently available evidence, intra-articular platelet-rich plasma (PRP) injection provides more superior pain relief, efficacious function improvement, and similar risk of adverse events when compared with HA injection and placebo in the treatment of KOA patients. However, we were not able to confirm the effect of other aspects of PRP on the treatment of KOA patients, including numbers of PRP injections (1 or ≥ 2), injection intervals (weekly or monthly), PRP spinning techniques (single or double), mean platelet concentration (> or < 5 × baseline), PRP category (LP-PRP or LR-PRP), and use an activator or not.

In the past few years, more and more researchers noticed the potential of PRP in the treatment of musculoskeletal diseases, such as rotator cuff tear, lateral epicondylitis, patellar tendinopathy, osteoarthritis, and Achilles tendon repair [19–26]. Given the properties of regenerative effect and anti-inflammatory potential in PRP, a number of researches [19, 23, 24, 27–35] explored the curative effect of intra-articular PRP injection in the treatment of patients with osteoarthritis, especially with knee osteoarthritis. However, in the current clinical guidelines of orthopedic surgeons, the use of PRP injection for KOA patients is uncertain [8, 37]. Few guidelines recommend PRP injection to treat KOA. This may be related to the different results reported in current high-quality evidence-based medical articles. Of the meta-analyses published in the last 3 years, only one article [31] considered PRP to have similar efficacy to HA, and other articles [30, 32, 33] suggested that PRP injection is more effective than HA in KOA patients.

Three meta-analyses [30, 32, 33] evaluated adverse events after PRP injection and HA or placebo in the treatment of KOA patients, including pain, stiffness, syncope, dizziness, headache, nausea, or infection. Shen et al. [33] indicated no severe complications were recorded and all adverse events were self-resolved in days. All of these articles suggested no statistical difference in adverse events between PRP injection and HA or placebo. Only one meta-analysis [30] compared the pooled effect sizes of primary outcomes with the minimum clinically important differences (MCID), which determinate whether significant outcomes have clinically meaningful implications [55, 56]. It demonstrated that compared with HA, PRP injection has better pain relief and function improvement in the 12 months follow-up (the CI of WOMAC pain and function scores was greater than the MCID), with no statistical difference in 6 months follow-up.

Riboh et al. [57] performed a network meta-analysis, which compared the clinical outcomes and adverse events between *LP-PRP*, *LR-PRP*, HA, and placebo. It included 6 RCTs (Level I) and 3 prospective comparative studies (Level II) and illustrated the effect of different leukocyte concentrations on PRP injection. This article suggested that *LP-PRP* has better functional outcome scores compared with HA and placebo in the treatment of KOA, with no difference between *LR-PRP* and HA. It also found no significant difference between PRP, HA, and placebo in adverse events and indicated leukocyte concentration may not directly relate to adverse events in PRP injection. In addition, unfortunately, we rarely found other scholars that compare the effects of different preparation methods, concentrations, and frequency of injection on the efficacy of PRP in the treatment of KOA. This is perhaps the focus of our future research.

The strengths of this summary of meta-analyses are based on the best currently available evidence to evaluate the clinical outcomes of PRP injection in the treatment of KOA patients. Three authors used these different appraisal tools [39–41] to assess the quality of each included meta-analysis, and each meta-analysis was Level I evidence.

### Limitations

There are also several limitations in this study. First, all included meta-analyses only evaluate the clinical outcomes of PRP injection at 6 months and 12 months follow-up, none of them was a median or long follow-up. Second, only one paper [30] performed a subgroup analyses of the different details of PRP injection, such as times of PRP injection, PRP spinning techniques, mean platelet concentration, PRP category, activation or not, and risk of bias. Therefore, it is not clear about the effect of the different details in PRP injection. Third, none of the included meta-analysis conducted a subgroup analysis about the OA grade (including Kellgren-Lawrence grade and Ahlback grade), so we do not understand which grade of OA can get more benefits from intra-articular PRP injection. Otherwise, heterogeneity is inevitable among the patients included in these meta-analyses, such as the age of patient, duration of knee pain before injection, sex, BMI, and so on. Finally, although a total of 1677 patients were included in this summary of meta-analysis, the included meta-analysis had included several primary articles which only contained a smaller sample size, and it may be a potential source of bias.

Therefore, more rigorous randomized controlled trials, which focus on a very specific question, such as which PRP spinning techniques, or which mean platelet concentration of PRP, or which frequency of injection of PRP can provide better clinical outcomes or which grade of OA can get more benefits from intra-articular PRP injection, are also needed to perform. Meanwhile, the articles with med-long-term follow-up are also needed to conduct and assess the curative effect of PRP injection.

## Conclusion

For short-term follow-up (≤ 1 year), intra-articular PRP injection is more effective in terms of pain relief and function improvement at short-term follow-up in the treatment of KOA patients than HA and placebo, and there is no difference in the risk of an adverse event between PRP and HA or placebo.

## Data Availability

All data are fully available without restriction.

## Abbreviations

HA: Hyaluronic acid
IA-HA: Intra-articular hyaluronic acid
IA-PRP: Intra-articular platelet-rich plasma
KOA: Knee osteoarthritis
LP-PRP: Leukocyte-poor PRP
LR-PRP: Leukocyte-rich PRP
PRP: Platelet-rich plasma
QUOROM: The Quality of Reporting of Meta-analyses
RCTs: Randomized controlled trials
